# Automated computer-based detection of encounter behaviours in groups of honeybees

**DOI:** 10.1038/s41598-017-17863-4

**Published:** 2017-12-15

**Authors:** Christina Blut, Alessandro Crespi, Danielle Mersch, Laurent Keller, Linlin Zhao, Markus Kollmann, Benjamin Schellscheidt, Carsten Fülber, Martin Beye

**Affiliations:** 1 Evolutionary Genetics, Heinrich-Heine University, Düsseldorf, Germany; 2 Biorobotics Laboratory (BioRob), École polytechnique fédérale de Lausanne (EPFL), Lausanne, Switzerland; 3 Neurobiology, MRC Laboratory of Molecular Biology, University of Cambridge, Cambridge, United Kingdom; 4 Department of Ecology and Evolution, Université de Lausanne, Lausanne, Switzerland; 5 Mathematical Modelling of Biological Systems, Heinrich-Heine University, Düsseldorf, Germany; 6 Faculty of Electrical Engineering & Information Technology, University of Applied Sciences, Düsseldorf, Germany

## Abstract

Honeybees form societies in which thousands of members integrate their behaviours to act as a single functional unit. We have little knowledge on how the collaborative features are regulated by workers’ activities because we lack methods that enable collection of simultaneous and continuous behavioural information for each worker bee. In this study, we introduce the Bee Behavioral Annotation System (BBAS), which enables the automated detection of bees’ behaviours in small observation hives. Continuous information on position and orientation were obtained by marking worker bees with 2D barcodes in a small observation hive. We computed behavioural and social features from the tracking information to train a behaviour classifier for encounter behaviours (interaction of workers via antennation) using a machine learning-based system. The classifier correctly detected 93% of the encounter behaviours in a group of bees, whereas 13% of the falsely classified behaviours were unrelated to encounter behaviours. The possibility of building accurate classifiers for automatically annotating behaviours may allow for the examination of individual behaviours of worker bees in the social environments of small observation hives. We envisage that BBAS will be a powerful tool for detecting the effects of experimental manipulation of social attributes and sub-lethal effects of pesticides on behaviour.

## Introduction

Honeybees, like other eusocial insects, form societies in which their members integrate their behaviours to form a single functional unit (often described as ‘superorganisms’)^1^. In honeybee colonies, for example, the brood is collectively reared by the worker bees under constant temperature conditions in worker-made and well-structured wax combs^2^. We still have little knowledge on how the collaborative features are regulated within the colony by single workers’ task engagements, worker-worker interactions and environmental cues.

A honeybee may engage in many behavioural tasks, for example, cell cleaning, brood feeding, comb building, pollen and nectar storing, and foraging^3^. The many in-hive tasks are usually performed within the first three weeks of their life, whereas foraging tasks are performed later^3^. Individual task engagements are flexible and are adapted according to the colony’s needs^4,5^. Differences in individuals’ internal response thresholds for task-specific stimuli (response threshold model)^6–8^, actively seeking for tasks (foraging for work model)^9^, repeatedly performing the same task when being successful at it (self-reinforcement models)^8,10^ and information transferred by social partners through direct contact^11^ may play an important role in the organisation of task engagements within the colony.

Gaining continuous behavioural information on each single worker, their direct contacts (encounters) to other worker bees and their interactions with the local environment would facilitate the further characterization of the underlying mechanisms of colony organization. However, we currently lack methods that enable the collection of simultaneous and continuous behavioural information for each individual worker bee in the environment of a colony^12^. In current methods, behaviours are manually detected by an observer either from video recordings of small observation hives or from direct observations^3,13–15^. These manually detected behaviours represent only a fraction of the behaviours that the many worker bees can display in a colony, especially when the behaviour is frequently performed, for example, in the case of encounter behaviours.

In honeybees, encounter behaviours between workers are characterized by the following: the two worker bees face each other head to head and their moving antennae are repeatedly in contact. Encounter behaviours summarize different worker-worker interaction behaviours that display constant antennal contact and can be further grouped into the following behaviours: (i) antennation behaviour, which is required to initialize and maintain a contact^16^, whereby the antennae of two worker bees are in constant contact but no other features of the following behaviours are displayed; (ii) begging behaviour, in which a worker bee begs for food from another nestmate;^16,17^ (iii) offering behaviour, in which a worker bee offers food to another nestmate;^17^ and, (iv) trophallaxis behaviour, in which nectar from the crop is exchanged between two bees^18,19^.

Worker bees may perform begging behaviour to gain information about the quality and source of nectar offered by the incoming forager bees^18,20–22^. Incoming forager bees perform offering behaviour to unload the collected nectar to a recipient in-hive worker bee via trophallaxis^20,23–25^. Returning foragers presenting high-quality nectar show increased offering behaviour as well as increased dancing behaviour^26^. They more often find a recipient bee and will more often return with nectar to the colony. Effects of different nectar qualities on worker-worker interaction establish a control mechanism for the workers’ foraging engagement, performance and the influx of high-quality nectar^27^. Despite their role in regulating workers’ foraging engagement and performance^23,28^, we have little knowledge on other possible roles that these encounter behaviours may have in task engagements and colony organization.

In this study, we introduce the Bee Behavioral Annotation System (BBAS), which enables the automated classification of worker-worker encounters within a group of honeybees. We obtained continuous information on workers’ positions and orientations over time by simultaneously tracking 100 bees tagged with a 2D barcode by adapting a tracking device that was developed for ants^29^. From this tracking information, behavioural and social features were computed, and a behaviour classifier was trained based on machine learning using the Janelia Automatic Animal Behavior Annotator (JAABA) program^30^. Our study demonstrates that we can automatically and accurately classify encounter behaviours within a group of bees. This system has the prospect of automatically obtaining quantitative and continuous behavioural information on hundreds of bees at once in small colonies.

## Results

### Automatic classification of encounter behaviours in a group of worker bees

To automatically classify worker behaviours in a small observation hive, we developed the BBAS. We obtained tracking information from individual worker bees in a small group and computed behavioural features (per-frame features), which were utilized to classify behaviours. Per-frame features represented parameters calculated from the tracking information that provided information on the bees’ behavioural properties in each frame. Such properties included, for example, a bee’s speed or orientation towards a nestmate (see Kabra *et al*.^30^ for a detailed listing of per-frame features). We applied the per-frame features to manually labelled behaviour classes to train a machine learning-based system and thus generate an automatic behaviour classifier.

**Figure 1:**
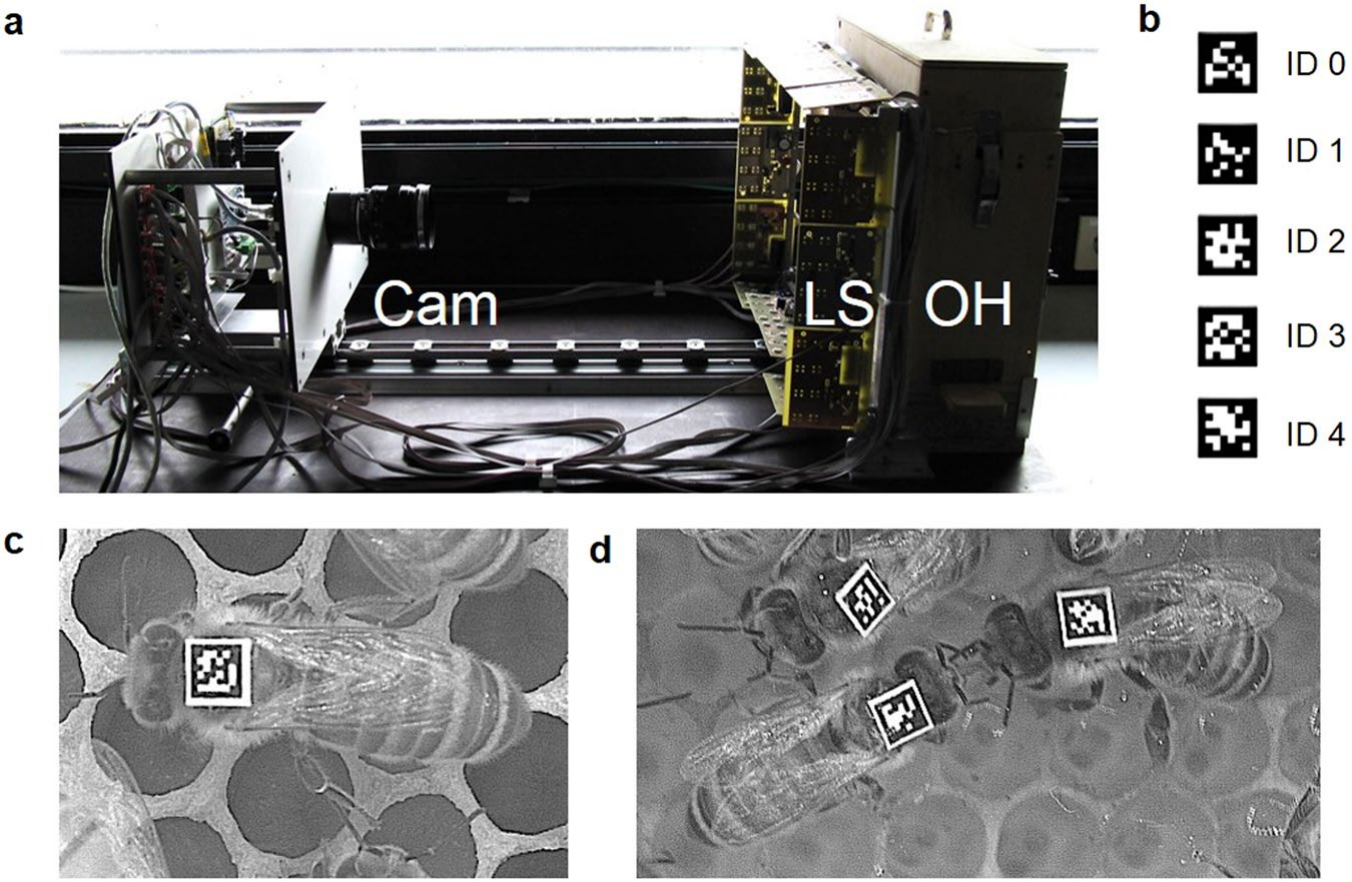
Setup of the tracking device. (**a**) The tracking device consisted of a high-resolution camera (Cam), an infrared lighting system (LS) and the observation hive holding one “Deutsch Normal” comb (OH). The entire device was placed under a cardboard box in a laboratory. (**b**) Examples of 2D barcodes from the AprilTags library. (**c**) Bee marked with a tag bearing a 2D barcode. (**d**) Encounter behaviour between two worker bees defined by the head to head orientation and the antennal contact of the interacting bees. This specific encounter shown is trophallaxis.

First, we adapted a tracking device developed for ants^29^ to obtain information on the position and orientation of individually tagged bees at a rate of four frames per second. In our setting, we tracked 100 newly emerged worker bees for two days. Bees were individually tagged with 2D barcodes from the AprilTags library^31^ printed on 2 × 2 mm tags and housed in a small observation hive on a single comb providing food (Fig. 1a–c). We chose a rate of four frames per second to ensure that we obtained sufficient information on the bees’ position and orientation for subsequent use in automatic behaviour classification. To test whether the chosen rate captured sufficient information we determined the average change in posiotion and orientation of bees (see Supplementary information online). On average, bees’ positions changed by 0.9 mm (SD ± 0.9 mm) from one frame to another, which corresponds to ~0.06% of an *Apis mellifera* worker size. Bees’ average change in orientation from one frame to another was 6° (SD ± 4°). These small changes in position and orientation suggest that we can capture sufficiently detailed information on the bees’ movements with the chosen rate. The AprilTag system was chosen because it is an actively maintained open source project and provides a robust system to minimize inter-tag confusion. It also has better performance on images taken under non-uniform lighting conditions as compared to several other similar systems^31^.

The results of the detection rate and positional accuracy of the tracking device of immobile tags glued to a comb and tags attached to moving and resting worker bees are summarized in Table 1. On average, resting bees were detected in 98.2% of the frames, whereas moving bees were detected in 90.8% of the frames. The orientation accuracy of immobile tags glued to a comb was 1.5° and the positional accuracy was 0.04 mm. The high detection rate and positional accuracy suggest that we can obtain a considerable amount of detailed information on the movement of each single worker in a group of bees.

**Table 1:** Detection rate and positional accuracy of the tracking device.

		No. of tracked tags	No. of frames analysed (sequence duration)^(3)^	Detection rate^(4)^ (%)	x/y position accuracy^(5)^ (mm ± SD)	Orientation accuracy^(5)^ (degrees ± SD)
Tags glued to a comb	immobile	100	1200 (5 min)	99.9	0.04^(6)^ ± 0.03	1.5 ± 0.8
Tags glued to a bee	resting^(1)^	10	240 (1 min)	98.2	n.d.^(7)^	n.d.^(7)^
	moving^(2)^	30	240 (1 min)	90.8	n.d.^(7)^	n.d.^(7)^

^(1)^Bee sits in one position without moving for ≥ 5 seconds.

^(2)^Bee walks across the comb without interacting with other bees, inspecting cells or performing any other task.

^(3)^Duration of the tracking.

^(4)^The percentage of frames in which tags were detected.

^(5)^Accuracy of the tracking device for the detected x/y centre position and the orientation.

^(6)^i.e., ~0.003% of an *Apis mellifera* worker size.

^(7)^Not determined (n.d.) because changes could result from the bees’ behaviours.

Second, to generate an automatic behaviour classifier, we computed per-frame features from the tracking information using the JAABADetect program^30^. Computing the per-frame features for the tracking information on 100 worker bees required a high-performance computing cluster. We used the social per-frame features to train a classifier for honeybee encounter behaviours^30^. The social per-frame features are a set of per-frame features providing information on an individual’s state in each frame in relation to its nearest nestmates. For example, the distance, orientation and speed towards another worker may be described by these features (see Kabra *et al*.^30^ for a detailed listing of social per-frame features).

Third, we determined whether we could automatically classify encounter behaviours between workers using an automatic behaviour classifier generated with the JAABA program. The automatic behaviour classifier was expected to classify the four different behaviours - antennation, begging, offering and trophallaxis - as a single class, which have the behavioural features of head to head orientation and antennal contact of two worker bees in common (Fig. 1d). To train the automatic behaviour classifier, we manually labelled 76 encounter behaviours and 77 non-encounter behaviours from 105 minutes of video recording and corresponding tracking information of the 100 tracked bees. We only labelled encounter behaviours of which we were highly confident that encounter behaviour was truly displayed. The 76 encounter behaviours (EBs) comprised a sample of 28 antennation, 8 begging, 6 offering and 34 trophallaxis behaviours (see Supplementary Videos V1-V4 online for examples of the four encounter behaviours). The non-encounter behaviours (NEBs) represented a sample of 46 sitting, 20 walking, 7 self-grooming, 1 social grooming and 3 sitting with subsequent walking behaviours. We trained the classifier by entering the 76 EBs and 77 NEBs (training set) bit by bit into the JAABA program in five training rounds until we observed no further improvement in the cross-validation estimates (see Supplementary information online for details on cross-validation). Cross-validation estimates were obtained by randomly splitting the EBs and NEBs from the training set into testing and training subsets. The training subset was used to train the classifier while the testing subset was used to subsequently estimate the classifier’s error rate on classifications^30^. Table 2 presents the final cross-validation estimates from 10 cross-validation rounds for our trained ‘encounter classifier’. The estimates represent the percentage of frames automatically classified as EB* and NEB* by the ‘encounter classifier’ (asterisks indicate automatically classified behaviours; see Supplementary information online for details on calculation of estimates). Of the EB frames, 77.3% were correctly classified by our ‘encounter classifier’ (SD ± 1.3%, Table 2), whereas 73.7% of the NEB frames were correctly classified (SD ± 1.2%, Table 2). The false positive rate was 26.3% (NEB frames falsely classified as EBs*), whereas the false negative rate was 22.7% (EB frames falsely classified as NEBs*; Table 2).

**Table 2:** The accuracy of the trained ‘encounter classifier’ estimated through cross-validation on the labelled frames for EBs and NEBs.

		Automatically detected by the ‘encounter classifier’	
		Encounter (EB*)^(6)^ (±SD) (%)^(2)^	Non-encounter (NEB*)^(6)^ (±SD) (%)^(2)^
Manually annotated^(1)^	Encounter (EB)	77.3 (±1.3)^(3)^	22.7 (±1.3)^(5)^
	Non-encounter (NEB)	26.3 (±1.3)^(4)^	73.7 (±1.2)^(3)^

^(1)^The manually labelled high-confidence behaviours (EBs and NEBs) used to train the classifier.

^(2)^Mean estimates with standard deviation (SD) of the 10 rounds of cross-validation. Estimate values are given as percentage of frames correctly or falsely classified as EBs or NEBs using the classifier.

^(3)^Frames correctly classified as EB or NEB (true positives).

^(4)^NEB frames falsely classified as EB* (false positives).

^(5)^EB frames falsely classified as NEB* (false negatives).

^(6)^Asterisks indicate automatically classified behaviours.

Next, we examined whether our classifier was able to correctly classify all 76 manually labelled EBs from our training set. Since the training set included the different behaviour classes - antennation, begging, offering and trophallaxis - we examined whether the classifier could correctly classify these four different behaviours as encounter behaviour. We determined the classification rate and observed that all manually labelled encounter behaviours of the training set were correctly detected by our classifier (training set in Table 3; Supplementary Table S1).

**Table 3:** Comparison of manually annotated behaviours (EBs and NEBs) and automatically classified behaviours (EBs* and NEBs*).

		Automatically detected by the ‘encounter classifier’	
		Encounter (EB*) (%)	Non-encounter (NEB*) (%)
Training set^(1)^	Encounter (EB)	100	0
	Non-encounter (NEB)	0	100
Testing set^(2)^	Encounter (EB)	93	7
	Non-encounter (NEB)	28^(3)^	n.d.^(4)^

^(1)^The manually labelled high-confidence behaviours (EBs and NEBs) used to train the classifier

^(2)^Manually annotated behaviours not used to train the classifier

^(3)^Automatically detected behaviours falsely classified as EB* by the ‘encounter classifier’

^(4)^not determined (n.d.) because we did not manually annotate NEBs for the testing set and thus could not determine the automatic classification rate.

We then determined the accuracy of our ‘encounter classifier’ by comparing manual annotations and automatic classifications of behaviours that were not included in our initial training set. We manually annotated 43 encounter behaviours comprising 4 trophallaxis, 8 begging, 12 offering and 19 antennation behaviours (testing set; see Supplementary Table S1). Our ‘encounter classifier’ detected 93% of the manually annotated encounter behaviours in this testing set. The false negative rate was 7%, whereas 28% of the automatically detected behaviours were falsely classified as EBs* (testing set in Table 3; Supplementary Table S1). We re-examined the falsely classified EBs* and found that 15% of the 28% falsely classified EBs* displayed similar features to those of encounter behaviours, i.e. head to head orientation and proximity of two bees. However, these falsely classified EBs* collectively lacked antennal contact. Of the behaviours falsely classified as encounters, 13% were unrelated to encounter behaviour, i.e. displayed no features characterizing encounter behaviours. The results on the high classification rates suggest that the BBAS can be used to automatically and accurately annotate encounter behaviours in groups of honeybees.

### Classification of trophallaxis behaviour based on the duration of the encounter behaviour

We demonstrated that we could automatically classify the different encounter behaviours, antennation, begging, offering and trophallaxis, as a single behavioural class with our ‘encounter classifier’. Next, we considered whether we could use the duration of the different encounter behaviours to distinguish these from each other. In 105 minutes of the 22 hours of video recording, we measured the frequency and duration of antennation, begging, offering and trophallaxis behaviours in the group of 100 worker bees.

**Figure 2:**
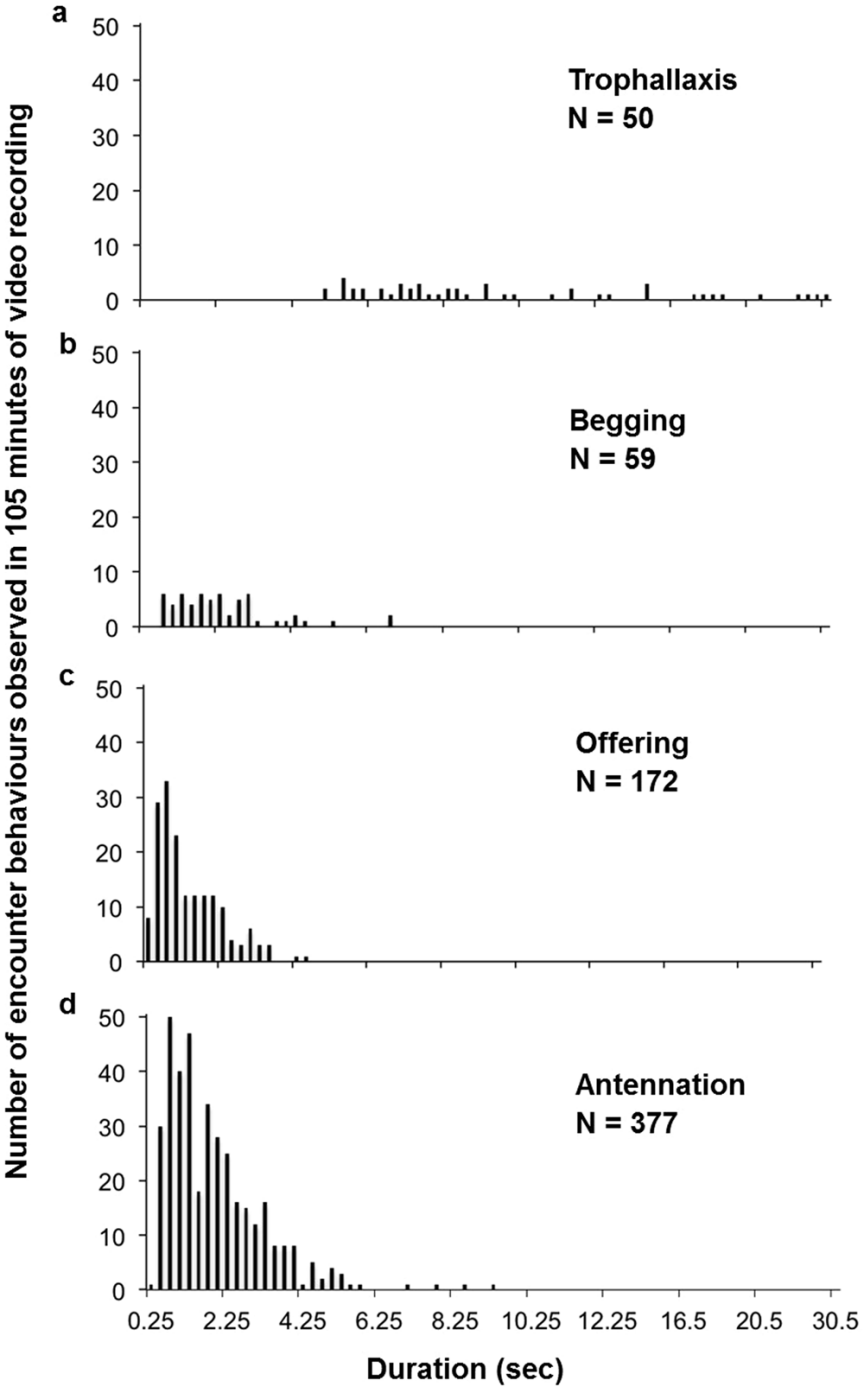
Number of encounter behaviours observed for the different duration of encounter behaviours from the four behaviour classes. (**a**) Trophallaxis, (**b**) Begging, (**c**) Offering, (**d**) Antennation.

We manually detected 658 encounter behaviours from which 57% were antennation behaviours, 26% were offering behaviours, 9% were begging behaviours and 8% trophallaxis behaviours (Table 4; Supplementary Videos V1-V4 online). The median duration of the trophallaxis behaviours was 8 seconds (75% percentile: 13 seconds; range of duration: 5–30.5 seconds; Table 4; Fig. 2a). The median duration of antennation, offering and begging behaviours was much shorter, ranging from 1 to 2 seconds with a considerable overlap in the 75% percentile (range of durations: antennation: 0.25–9.25 seconds, offering: 0.25–4.5 seconds, begging: 0.75–6.75 seconds; Table 4; Fig. 2b-d). There was a significant difference between the duration of the four different encounter behaviours (One Way ANOVA on Ranks: N = 658, α = 0.05, H = 175, d.f. = 3, P =  < 0.001). Post hoc tests showed that pairwise comparisons were significantly different except for begging vs. antennation behaviours (Dunn’s Method, α = 0.05: trophallaxis vs. offering: N = 222, Q = 13, P < 0.001; trophallaxis vs. antennation: N = 427, Q = 10.7, P < 0.001; trophallaxis vs. begging: N = 109, Q = 6.7, P < 0.001; begging vs. offering: N = 231, Q = 5.3, P < 0.001; antennation vs. offering: N = 549, Q = 5.2, P < 0.001; begging vs. antennation: N = 436, Q = 2.3, P = 0.138). This result suggests that the duration of encounter behaviours could be utilized to distinguish the different encounter behaviours from each other.

**Table 4:** Frequency and duration of the different manually detected encounter behaviours.

Encounter behaviour	No. of encounters	Relative proportion (%)	Min. duration (sec)	Max. duration (sec)	Median (sec)	75% percentile (sec)
Antennation	377	57	0.25	9.25	1.8	2.5
Offering	172	26	0.25	4.5	1	1.9
Begging	59	9	0.75	6.75	2	3
Trophallaxis	50	8	5	30.5	8.4	12.9

Next, we tested whether encounter behaviours could be accurately classified as antennation, begging, offering or trophallaxis based solely on their duration. Therefore, we analysed the ranges of duration of the 658 encounters from the four behaviour classes to determine whether duration thresholds could be used as classifier for the different encounter behaviours. Hereby, we attempted to find thresholds above which behaviours could be reliably classified as one of the four behaviour classes. We observed that duration thresholds could not be utilized as classifiers for begging, offering and antennation behaviours since their ranges of duration overlapped too strongly (Table 4; Fig. 2). When considering only behaviours with duration of 5 and more seconds, we observed that all trophallaxis behaviours could be correctly classified (100%; Table 5). Non-trophallaxis behaviours (i.e. begging and antennation behaviours), however, were falsely classified as trophallaxis behaviours with a false positive rate of 8% (Table 5).

**Table 5:** The classification of trophallaxis behaviours of manually detected and automatically detected encounter behaviours using the duration threshold of ≥ 5 seconds.

	Manually classified by duration among the 658 manually detected behaviours^(1)^		Automatically classified by duration among the EBs* from the testing set^(2)^	
	Trophallaxis (%)^(3)^	Non-trophallaxis (%)^(3)^	Trophallaxis* (%)^(4)^	Non-trophallaxis* (%)^(4)^
Trophallaxis^(5)^	100	0	100	0
Non-trophallaxis^(5)^	8	92	28	72

^(1)^We manually classified trophallaxis behaviours from the 658 manually detected encounter behaviours using the duration threshold of ≥ 5 seconds.

^(2)^We applied the ‘encounter classifier’ with the duration threshold of ≥ 5 seconds to the 43 manually annotated encounter behaviours not used for training.

^(3)^Percentage of the manually detected trophallaxis and non-trophallaxis behaviours that were manually classified as trophallaxis using the duration threshold of ≥ 5 seconds.

^(4)^Percentage of the manually annotated trophallaxis and non-trophallaxis behaviours from the testing set that were automatically classified as trophallaxis* and non-trophallaxis* (asterisks indicate automatic classification) using the duration threshold of ≥ 5 seconds.

^(5)^Trophallaxis and non-trophallaxis behaviours that were manually annotated by the observer.

We then tested whether trophallaxis behaviours could be automatically classified based on their duration. We applied the duration threshold of ≥ 5 seconds to the automatically classified EBs* from the testing set comprising 43 encounter behaviours. We observed that 100% of the trophallaxis behaviours were automatically classified (Table 5). However, 28% of the detected behaviours were falsely classified as trophallaxis (false positive rate; Table 5). These classification rates suggest that we can automatically classify the vast majority of trophallaxis behaviours in a group of worker honeybees using our ‘encounter classifier’ together with the duration threshold of ≥5 seconds.

## Discussion

We introduced the BBAS, a system that can automatically classify stereotypical behaviours of individual workers in a group of honeybees. Our results show that the BBAS can be reliably used to automatically detect encounter behaviours.

Current behavioural observation methods usually require the manual detection of behaviours by an observer^12^. Manual detection limits the number of observable behaviours, especially when the behaviour is frequently displayed by the many worker bees in a colony, as is the case for encounter behaviours. In this study, we accurately classified encounter behaviours between worker bees using automatic classification. Of the encounter behaviours that were manually annotated, 93% were accurately detected. Even though our classifier may not detect 7% of the encounter behaviours, the large number of behaviours of the many worker bees that can be detect over multiple days of observation produces a reliable test sensitivity. This statistical power will support the identification of even tiny differences between internal physiological states or the effects of experimental manipulation. According to the manual annotations, our classifier falsely classified other behaviours as encounter behaviours. Of these false detections, 13% had no similarity to encounter behaviours, whereas 15% had a close similarity to encounter behaviours, possibly suggesting that our classifier can detect a broader spectrum of encounter and encounter-related behaviours than can be manually annotated. These borderline cases may have a similar biological function and require further investigation.

In this study, the duration of the four different classes of encounter behaviours – trophallaxis, begging, offering and antennation - was obtained from 100 same-aged bees kept in a one-frame observation hive without a queen and brood. Our results showed that trophallaxis behaviours lasted between 5 and 30.5 seconds. The duration of offering and begging behaviours ranged from 0.25 to 6.75 seconds while antennation lasted 0.25 to 9.25 seconds. These measurements correspond to previous reports on the duration of trophallaxis, begging and offering behaviour that were obtained under more natural conditions (queenright colonies in one - or two-frame observation hives^17,19,26^). Trophallaxis behaviours of different aged worker bees in these small queenright colonies lasted 4 to 30 seconds while begging and offering lasted less than 0.5 to 10 seconds^17,19,26^. This constancy under different conditions suggests that duration can possibly be used as a predictive parameter to distinguish among the behavioural classes of encounters.

Our survey of manually annotated encounter behaviours suggests that a duration threshold of ≥ 5 seconds for an encounter behaviour can be used to accurately separate trophallaxis behaviour from the other encounter behaviours (begging, offering and antennation). When we applied our ‘encounter classifier’ together with the duration threshold, we were able to classify 100% of the manually annotated trophallaxis behaviours. However, the false positive rate was relatively high (28%), suggesting that we may need further adjustments of the behaviour duration parameter to reduce false classifications.

It has been proposed that encounter behaviours and the transmission of food are ways for worker bees to gather information about their colony’s state and thus can adjust their behaviours according to the colony’s needs^32–35^. So far, we have detailed knowledge on the role of trophallaxis, begging and offering behaviours between incoming foragers and worker bees inside the colony in accessing information about the quality and source of nectar and the honey stores of the colony. Foraging worker bees usually unload the nectar from the honey crop to the in-hive worker bees via trophallaxis^18,23,36^. The recipient worker bees store the nectar within the wax cells or further reduce the water content. Offering behaviour is performed by the returning nectar foragers, which are willing to unload their crop contents to a recipient worker bee^17^. Inside the nest, worker bees beg incoming forager bees to receive nectar^17,22,23,37^. The rate of begging behaviour is affected by the colony’s state and the amount of stored honey in the colony^38^. Antennation behaviour is essential in making and maintaining the contact between encountering bees^16,20^. We envisage that with more classifiers trained for other behaviours, we can further examine the possible effects of encounter behaviours on subsequent task engagement.

For training the classifier and for measuring the accuracy of detection, we used 100 tagged worker bees in this study. However, with the current setup the BBAS can track up to 1000 worker bees on a brood comb in a small observation hive (preliminary data). It can be further scaled up to over 2000 worker bees by adding an additional camera, lighting system and cluster of five computers. Hence, we suggest that the BBAS will enhance our ability to gather knowledge on worker bees’ individual and collective behaviours. With more classifiers trained to detect different behaviours in honeybees, the BBAS can be used to examine single-worker behavioural phenotypes and worker-worker interactions within small observation hives. We envisage that the BBAS will be a powerful tool to detect the experimental effects of genetic and physiological manipulations on single workers^39,40^. Additionally, we propose that the BBAS can be an accurate method for measuring the sub-lethal effects of pesticides on behaviour^41^. The key to understanding the effects of pesticides on honeybee colonies is gaining knowledge on how these influence individual behaviour. With the BBAS we will be able to analyse the effects of pesticides on individual behaviour because we can continuously and simultaneously quantify the in-hive behaviours of hundreds of worker bees under standardized conditions with computer-based classifiers. For encounter behaviours, for example, we can analyse the effects of pesticides on the duration of encounters or their quantity.

In conclusion, we foresee that the BBAS will be beneficial in various research areas for honeybee researchers who need to obtain detailed behavioural information of hundreds of individual bees.

## Methods

### Tracking device and procedure

Video recordings of worker bees on a comb and tracking information were obtained with a tracking device that was developed for ants by Mersch *et al*.^29^ and modified for tracking honeybees (see Supplementary information online). The honeybee tracking device consisted of a monochrome high-resolution camera, a cluster of five desktop computers, an infrared lighting system and an observation hive holding a single “Deutsch Normal” comb (Fig. 1a). The infrared light was provided in flashes synchronized with the images taken every quarter second (4 frames per second) by the camera. To omit daylight exposure, both the observation hive and the camera stood in a laboratory covered by a cardboard box that was lined with infrared-reflecting foil, which intensified the infrared illumination of the comb area. The cardboard box was equipped with a ventilation device that kept the temperature at approximately 29 °C (±1 °C).

We used 2 × 2 mm tags bearing 2D barcodes from the AprilTags library (Fig. 1b)^31^ to tag and track honeybee workers. These 2D barcodes consisted of a square outline with a 36-bit code word encoded in the interior, which could generate up to 2320 unique identification (ID) numbers. An experiment on mortality and behavioural observations of tagged bees showed that bees bearing tags survived and behaved as untagged bees did (see Introductory experiments and observations in Supplementary information online). The tracking information obtained by the tracking software^29^ contained (after postprocessing) the tag’s ID number, the x- and y-coordinates of its centre and its orientation with the corresponding frame number and timestamp in UNIX time (with a precision of 1/100 seconds).

### Automatic behaviour classification using the tracking information

From the tracking information, we used the JAABADetect program^30^ to compute social per-frame features to provide information on the bees’ properties in relation to their nearest nestmate in each frame (for example, the distance, speed, and orientation to the closest bee; see Kabra *et al*.^30^ for a detailed listing of social per-frame features).

To produce the ‘encounter classifier’, we labelled examples of encounter and non-encounter behaviour in 105 minutes of tracking information and video material using the graphical user interface of the JAABA program^30^. We only labelled encounter and non-encounter behaviours for which we had high confidence in classification. Thus, for encounter behaviours we only labelled those for which we could confidently identify that behavioural features characterizing encounter behaviours were displayed. Information about the social per-frame features that were computed from the tracking information was used to train the ‘encounter classifier’ via machine learning implemented in the JAABA program^30^.

The classifier’s accuracy was determined using the cross-validation method implemented in the JAABA program^30^. We used JAABA’s default settings for the cross-validation and performed 10 cross-validation rounds to obtain an average estimate on the classifier’s accuracy (see Supplementary information online for more details on calculation of accuracy and cross-validation).

### Manual annotation of encounter behaviours and further analysis

We manually examined the video recordings to detect all encounter behaviours. We determined the duration in seconds and the type of encounter behaviour: i) antennation behaviour, ii) begging behaviour, iii) offering behaviour, iv) trophallaxis behaviour.

Statistical analyses were performed using the SigmaPlot 13 software.

### Bee handling

We used newly emerged honeybees that originated from a colony of western honeybee *Apis mellifera* from our bee yard at the Heinrich-Heine University of Düsseldorf, Germany. A sealed brood comb was taken from the source colony and incubated at 34 °C. Emerging worker bees were collected when they were 0–24 hours old. One hundred bees were marked with hand-cut tags by gluing these centrally on the thorax of the bees with glue (“Opalith Zeichenleim”, Heinrich Holtermann KG, Brockel, Germany).

The bees were tracked from May 3^rd^ to May 4^th^, 2016 on a comb comprising 40 capped cells filled with honey. Bees were restricted to one side of the comb without a queen. As worker-worker encounters were the interest of this study, neither a queen nor drones were included in the group. The comb did not contain brood because we used newly emerged worker bees for tracking, and it is known that brood rearing first begins at an age of two to three days^3,24^.

To ensure that sufficient encounter behaviours occurred during the tracking process, a proportion of the bees were either fed ad libitum with a sugar solution (Ambrosia Bienenfutter-Sirup, Nordzucker AG, Braunschweig, Germany) or starved before tracking was started. On the first day of tracking, 16 bees were fed with the sugar solution before starting the tracking experiment, whereas the remaining bees were starved for approximately an hour. For sustenance, we provided the bees with a sugar pastry (Apifonda Futterteig, Südzucker AG, Mannheim, Germany) two hours after tracking was started. On the second day of tracking, we removed the sugar pastry and fed 15 of the 100 bees again with the sugar solution. The other 85 bees were starved for three hours. The 15 bees were reintroduced into the observation hive before tracking began. In total, information from 22 hours of tracking was generated for 96 bees. Four bees lost their tags during tracking.

### Data availability

The datasets generated and analysed during the current study are available from the corresponding author on reasonable request. Programs developed for this study will be shared and can be requested from the corresponding author.

## Electronic supplementary material

Supplementary information

Supplementary Video V1

Supplementary Video V2

Supplementary Video V3

Supplementary Video V4
