# Supplementary material for: Automated computer-based detection of encounter behaviours in groups of honeybees
Source: Sci Rep. 2017 Dec 15;7:17663. doi: 10.1038/s41598-017-17863-4 (PMC5732155; doi:10.1038/s41598-017-17863-4)
Supplement: Supplementary file 1 — Supplementary information [file 41598_2017_17863_MOESM1_ESM.pdf]

Supplementary information

Automated computer-based detection of encounter behaviours in groups of honeybees

Christina Blut[1,*], Alessandro Crespi[2], Danielle Mersch[3], Laurent Keller[4], Linlin Zhao[5], Markus Kollmann[5], Benjamin Schellscheidt[6], Carsten Fülber[6], and Martin Beye[1,*]

[1]Evolutionary Genetics, Heinrich-Heine University, Düsseldorf, Germany; [2]Biorobotics Laboratory (BioRob), École polytechnique fédérale de Lausanne (EPFL), Lausanne, Switzerland; [3]Neurobiology, MRC Laboratory of Molecular Biology, University of Cambridge, Cambridge, United Kingdom; [4]Department of Ecology and Evolution, Université de Lausanne, Lausanne, Switzerland; [5]Mathematical Modelling of Biological Systems, Heinrich-Heine University, Düsseldorf, Germany; [6]Faculty of Electrical Engineering & Information Technology, University of Applied Sciences, Düsseldorf, Germany. *blut@hhu.de, martin.beye@uni-duesseldorf.de

Supplementary Table S1: Comparison of manual annotations (EBs) and automatic classifications (EBs*) among the different classes of encounter behaviours

	No. of behaviours from the training set		No. of behaviours from the testing set			
Classes of encounter behaviours	Manually annotated EBs [1)]	Automatically classified EBs* [2)]	Manually annotated EBs [3)]	Automatically classified EBs* [4)]		
				Correctly classified	False negatives [5)]	False positives [6)]
Trophallaxis	34	34	4	4	0	17
Begging	8	8	8	8	0	
Offering	6	6	12	12	0	
Antennation	28	28	19	16	3	

[1)] The manually labelled high-confidence behaviours (EBs) used to train the classifier (training set)
[2)] EBs* correctly classified by the trained 'encounter classifier'. A confidence threshold of > 0 was set for automatic classification; thus, only EBs with frames displaying confidence values above 0 were classified as EBs*
[3)] Manually annotated EBs not used to train the 'encounter classifier'
[4)] EBs* correctly classified by the trained 'encounter classifier'. A confidence threshold of > 0.2 was set for automatic classification; thus, only EBs with frames displaying confidence values above 0.2 were classified as EBs*
[5)] EBs falsely classified as NEBs* by the 'encounter classifier'
[6)] NEBs falsely classified as EBs* by the 'encounter classifier'

Supplementary Methods

Tracking device

Video recordings of worker bees on a comb and tracking information were obtained with a tracking device that was developed for ants by Mersch et al.[1]. For honeybee tracking, the following modifications were made to the tracking device: We used a monochrome high-resolution camera (hr29050MFLGEA GigE Compact CCD-camera, 6576x4384 pixels, SVS-VISTEK GmbH, Seefeld, Germany) equipped with an 50 mm F/2.0 camera lens (ZEISS Objektiv Makro-Planar T* 2.0/50 ZF, Carl Zeiss AG, Oberkochen, Germany) and an infrared-transmitting filter that was transparent to wavelengths starting from 780 nm (Heliopan RG780 Infrared Filter, Heliopan Lichtfilter-Technik Summer GmbH & Co KG, Gräfelfing, Germany). The tracking software was run on a cluster of five desktop computers (2x Intel Xeon E5-1620, 3.70 GHz, 16GB RAM, Rombus, Büren, Germany; 1x Intel Xeon E5-1630, 3.70 GHz, 16GB RAM, 1x Intel Core i5-3330, 3.00 GHz, 12GB RAM, 1x Intel Core i7-4790, 3.60 GHz, 8GB RAM, 1x Intel Xeon E5-1630, 3.70 GHz, 16GB RAM, Wortmann AG, Hüllhorst, Germany) that were connected through a Gigabit Ethernet switch (D-Link DGS-1016D Gigabit 16-Port Switch, D-Link GmbH, Eschborn, Germany). The camera was connected to the cluster via two Ethernet cables and took images of the surface of a single “Deutsch Normal” comb that was placed in an observation hive between two Plexiglas panels. The distance between the Plexiglas panels and the comb was about 12 mm (for more information see Introductory experiments and observations). To omit daylight exposure, both the observation hive and the camera were covered by a cardboard box in the laboratory. The box was lined with infrared-reflecting foil (LEE Farbfolie 273, Eckert Bühnenlicht, Wuppertal-Langerfeld, Germany). The infrared-reflecting foil intensified the infrared illumination of the comb area. The cardboard box was equipped with a ventilation device that kept the temperature at approximately 29°C (± 1°C). The lighting system consisted of fourteen custom-made electronic boards each equipped with six infrared light emitting diodes. The boards were arranged directly around the observation hive to avoid light reflections on the Plexiglas (Fig. 1a). The infrared light was provided in 5 ms flashes with a peak wavelength at 850 nm (outside of the bees’ visible range[2]) that were synchronized with the images taken every quarter second (4 frames per second). We used infrared light flashes rather than continuous infrared light since flashes had the advantage of producing less heat and thus facilitated temperature control under the cardboard box. Also, motion blur of fast moving bees was decreased because light flashes permitted us to use a higher light intensity and thus a reduced exposure time.

Tracking procedure

The tracking software and image processing were as described in Mersch et al.[1], except for the following modifications: Among the various 2D barcode sets provided by the AprilTags library, we used the 36h10 set (Fig. 1b), which

provides up to 2320 unique 2D barcodes. Each barcode is encoded as a unique 36-bit code word having a minimum Hamming distance (i.e. number of differences when comparing bit-by-bit with another code) of 10 bits to all the other barcodes[3]. Tags bearing the 2D barcodes were 2x2 mm in size, printed on waterproof white polyester foil (laserFOL.135 a4, matte surface, 135 µm, Papier & mehr, Neuenhaus, Germany) with a laser printer and cut out by hand. The tags were filmed by the camera at a resolution of 17 pixels/mm. During processing, the images were divided into 96 segments by the tracking software because it can only handle small images. Image segments overlapped by 100 pixels to ensure that tags located on segment borders were detected. Images were saved as video (AVI files) after they were resized to 1644x1096 pixels and compressed with the Xvid codec. The tracking software output the ID number, the x- and y-coordinates of the four corners of each tagged bee's tag with the corresponding timestamp in UNIX time (with a precision of 1/100 seconds) and the image number, and this information was stored in a comma-separated values (CSV) data file. This CSV data file was postprocessed to obtain the tag's centre and its orientation and to generate a binary data file containing the tag's ID number, its x- and y-coordinates of its centre and its orientation with the corresponding frame number and timestamp in UNIX time (with a precision of 1/100 seconds). Finally, to obtain the final binary data file, we processed the angle difference between the front of the tag and the front of the bee using a program from Mersch et al.[1] to ensure that the orientation given in the final data file represents the front of each bee (see Supplementary Fig. S2).

To estimate whether we can capture sufficient information on bees' position and orientation with a tracking rate of four frames per second, we measured the average change in x/y position and orientation of bees in four consecutive frames. We randomly chose 10 bees moving across the comb, calculated the average change in x/y position and orientation for each bee and then calculated the average change in x/y position and orientation with its standard deviation for all bees.

We examined the accuracy of our tracking device by determining the average detection rate of immobile tags (glued to a comb) and tags glued to bees. To measure the detection rate of the immobile tags, we distributed 100 immobile tags on a wax foundation positioned in a "Deutsch Normal" frame and tracked the tags for five minutes. To measure the detection rate of the tags on bees, we randomly selected 30 moving and 10 resting worker bees from three different video sequences and tracked them for one minute. For each tag, we calculated the percent of frames in which the tag was detected, and from this we calculated the average percent of detected frames for immobile tags and tags glued to bees. For the immobile tags, we also determined the accuracy for the detection of the x/y position and orientation. For both the position and orientation, we calculated the average difference between the tracking information of consecutive frames with its standard deviation.

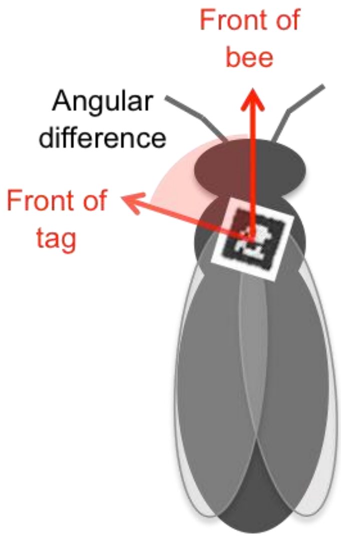

Supplementary Figure S2: Angle difference between the front of the tag and the front of the bee. If the front of the tag did not align with the front of the bee, we corrected the angle difference to ensure that orientations given in the tracking information corresponded to the bee's front.

Automatic behaviour classification using the tracking information

From the tracking information, we computed social per-frame features that provide information on the bees' properties in each frame in relation to its nearest neighbour (for exampe, the distance, speed, and orientation to the closest bee). We used the JAABADetect program[4] that was run on a high performance-computing cluster at the Heinrich-Heine University Düsseldorf, Germany. The JAABADetect and JAABA program were run in MATLAB version 8.3.0.532 (R2014a; The MathWorks GmbH, Ismaning, Germany). To obtain tracking data for the classifier's training procedure, we extracted three 15-minute and two 30-minute sequences of the total tracking data and video recordings using a program from Mersch et al.[1] and the free video tool VirtualDub (VirtualDub-1.9.11, virtualdub.org). From these data, we generated MATLAB-compatible .mat files using an in-house MATLAB application. This application also performed the following steps: first, if the tracking information in a single frame of a worker's trajectory was missing, the information was extrapolated from the mean of data points before and after the gap; second, trajectory segments representing missing information from more than 6 frames were excluded from the further analysis; third, trajectories shorter than 10 frames (for 15-minute sequences) and shorter than 100 frames (for 30-minute sequences) were not inherited by the MATLAB .mat file.

To train the 'encounter classifier', we first labelled examples of encounter and non-encounter behaviours using the video recordings and the graphical user interface of the JAABA program[4]. We only labelled encounter behaviours and non-encounter behaviours for which we had high confidence in classification. For encounter behaviours, we thus only labelled encounters for which we could confidently identify that behavioural features characterizing encounter behaviours were displayed. For automatic classification of encounter behaviours, we applied the hysteresis method which is a postprocessing tool implemented in the JAABA program (http://jaaba.sourceforge.net/Training.html#PostProcessing). This tool allows for reduction of falsely classified behaviours by setting a confidence threshold. Automatically classified behaviours are discarded if their confidence values lie below the set confidence threshold. Most falsely classified behaviours had low confidence values, thus by setting confidence thresholds these falsely classified behaviours could be reduced significantly. To determine confidence thresholds for the different data sets analyses in this study, we examined the classifier's positive and false positive classification rates for each data set with different confidence thresholds. We chose the confidence threshold providing the lowest rate of false classifications. We applied a confidence threshold of > 0 (i.e. no threshold) for the training set (EB) and a confidence threshold of > 0.2 for the behaviours not used for training. For the automatic classification of trophallaxis behaviours using our 'encounter classifier' with the duration threshold of ≥ 5 seconds, we applied a confidence threshold of > 0.4.

Training was performed with the social per-frame features computed from the tracking information (see the detailed list of social per-frame features in the Supplementary information of Kabra et al.[4]). We performed cross-validation to measure the classifier's accuracy using JAABA's default settings[4]. For cross-validation, the training set consisting of the labelled EBs and NEBs was spilt into subsets. By default JAABA does 7-fold cross-validation, thus the training set was split into 7 subsets. Hereby, 1/7 of the EBs and NEBs were assigned to a testing subset while 6/7 were assigned to a training subset. Later was used to train the classifier whereas the classifier's error rate was estimated by testing its accuracy on the testing subset[4]. The accuracy is tested by quantifying how many frames of the manually labelled EBs and NEBs were predicted on correctly or incorrectly by the 'encounter classifier'. Therefore, the number of frames manually labelled as EBs and NEBs and automatically predicted on as EB* and NEB* by the 'encounter classifier' are compared (asterisks indicate automatically classified behaviours). This results in a percentage value for frames correctly and falsely classified as EB* and NEB* by the 'encounter classifier'. The assignment of EBs and NEBs to either the testing or training subset is done randomly. Thus, the EBs and NEBs maybe part of a different subset for each cross-validation round performed. The estimated accuracy of the classifier may therefore

slightly vary for each cross-validation round. We performed 10 cross-validation rounds to obtain an average estimate of the classifier's classification accuracy.

Manual annotation of encounter behaviours and classification of trophallaxis
We manually examined the video recordings to detect encounter behaviours using the video tool VirtualDub (VirtualDub-1.9.11, virtualdub.org) with a software component from Mersch et al.[1] that enables the visualization of the bee's ID number in the video recording. We noted all encounters with the corresponding behaviour duration in seconds and determined the type of encounter behaviours: i) begging behaviour, ii) offering behaviour, iii) trophallaxis behaviour and iv) antennation behaviour. The different encounter behaviours are characterized by the following behavioural features: i) Begging bees tilted their head upwards, opened their mandibles and outstretched their proboscis[5] towards the mouthparts of the other bee[6]. The begging bee moved its antennae more or less intensely and oriented them towards the other bee. Additionally, the begging bee could show a grasping movement with its front legs[5]. ii) A bee displaying offering behaviour opened its mandibles, but it did not show tilting of its head or the outstretching of its proboscis (as observed for begging bees). The antennae were held low and close to the head[5]. iii) During trophallaxis behaviour the antennae of the bees were in contact, whereas the receiving bee outstretched its proboscis towards the mouthparts of the donating bee[5]. In addition to the behavioural features, we used the duration (> 4 seconds) of encounter behaviour to characterize trophallaxis behaviours and to differentiate them from the three other encounter behaviours because different studies have shown that an effective food transfer requires a contact of at least 4 seconds[7,8]. iv) Antennation behaviour was noted if two bees stood facing each other with their moving antennae in contact and no other behavioural features as described for the other three classes were shown[9-12].

Statistical analyses were performed using the SigmaPlot 13 program.

Introductory experiments and observations
Experiments and observations were performed prior to the tracking experiments in this study. We used newly emerged honeybees that originated from colonies of western honeybee *Apis mellifera* from our bee yard at the Heinrich-Heine University of Düsseldorf, Germany. Bees from the same experiment were of the same age. Sealed brood combs were taken from the source colonies and incubated at 34°C. Emerging worker bees were collected when they were 0–24 hours old and marked either with tags bearing 2D barcodes (2D barcodes were printed on waterproof foil) or a coloured dot on the thorax using a marker (POSCA Zeichenstift "Europa", Heinrich Holtermann KG, Brockel, Germany).

First, behavioural observations of bees marked with tags were conducted to determine if the tags affect their behaviour. We introduced 150 tagged bees into a queenright colony of a two-frame observation hive.

Observations were conducted from June 14th to July 26st, 2013 at the Heinrich-Heine University of Düsseldorf, Germany. We observed the bees' behaviour every second day for two hours. Tagged bees performed all in-hive tasks equally than their non-tagged nestmates. They entered comb cells and also left the hive for foraging trips returning with pollen loads.

Second, from July 8th to August 8th, 2013 we tested the mortality of bees marked with tags. We introduced 100 bees tagged with 2D barcodes and 100 bees marked with a coloured dot on their thorax into a queenright colony housed in a one-story beehive box. The hive stood in a flight cage. Bees were provided with pollen (Echter Deutscher Spezial Blütenpollen, Werner Seip – Biozentrum GmbH & Co. KG, Butzbach, Germany), sugar solution (Ambrosia Bienenfutter-Sirup, Nordzucker AG, Braunschweig, Germany) and water that they could forage for ad libitum. Over the period of the experiment, we counted the dead bees each day by searching the floor of the flight cage for marked bees. There was no difference in the survival of bees marked with tags and coloured dots (Log-rank test: $N = 200$, $\chi^2 = 2.7$, d.f. = 1, $P = 0.1$).

Third, when constructing our observation hive for the tracking experiments we started off with a distance of about 8 mm between the Plexiglas panels and the comb. This distance corresponds to the common distance of 8 mm between combs in a beehive box. We ascertained, however, that this distance was too narrow. First, this distance affected drone behaviour because they got stuck between the two surfaces. Second, the narrow space caused water to condense on the Plexiglas, which affected visibility. We thus increased the distance between the glass and the comb by 4 mm resulting in a distance of 12 mm.

Supplementary Video V1: Antennation behaviour. The highlighted bees with the ID numbers 2296 and 2302 (green) show antennation behaviour. The bees face each other with their moving antennae in contact.

Supplementary Video V2: Begging behaviour. The bee highlighted in red with the ID number 2276 (green) shows begging behaviour towards the bee highlighted in blue with the ID number 2261 (green). The begging bee (red) orientates its antennae towards the other bee, tilts its head upwards and grasps the other bee with its front legs.

Supplementary Video V3: Offering behaviour. The bee highlighted in red with the ID number 2302 (green) shows offering behaviour towards the bee highlighted in blue with the ID number 2045 (green). The offering bee (red) holds its antennae low and close to its head and does not tilt its head upwards as a begging bee would.

Supplementary Video V4: Trophallaxis behaviour. The highlighted bees with the ID numbers 2134 (green) and 2279 (green) show trophallaxis

behaviour. The antennae of both bees are in contact and the bee highlighted in red outstretches its proboscis towards the other bee.

References

1 Mersch, D. P., Crespi, A. & Keller, L. Tracking individuals shows spatial fidelity is a key regulator of ant social organization. *Science* **340**, 1090-1093, https://doi.org/10.1126/science.1234316 (2013).

2 Backhaus, W. Color opponent coding in the visual-system of the honeybee. *Vision Res* **31**, 1381-1397, https://doi.org/10.1016/0042-6989(91)90059-E (1991).

3 Olson, E. AprilTag: A robust and flexible visual fiducial system. *Proceedings of the IEEE International Conference on Robotics and Automation (ICRA)*, https://doi.org/10.1109/ICRA.2011.5979561, 3400-3407 (2011).

4 Kabra, M., Robie, A. A., Rivera-Alba, M., Branson, S. & Branson, K. JAABA: interactive machine learning for automatic annotation of animal behavior. *Nature Methods* **10**, 64-67, https://doi.org/10.1038/nmeth.2281 (2013).

5 Korst, P. J. A. M. & Velthuis, H. H. W. The nature of trophallaxis in honeybees. *Insect Soc* **29**, 209-221, https://doi.org/10.1007/Bf02228753 (1982).

6 Free, J. B. A study of the stimuli which release the food begging and offering responses of worker honeybees. *Br J Anim Behav* **4**, 94-101, https://doi.org/10.1016/S0950-5601(56)80129-9 (1956).

7 Farina, W. M. & Wainselboim, A. J. Thermographic recordings show that honeybees may receive nectar from foragers even during short trophallactic contacts. *Insect Soc* **48**, 360-362, https://doi.org/10.1007/Pl00001791 (2001).

8 Kühnholz, S., Seeley, T.D. The control of water collection in honey bee colonies. *Behav Ecol Sociobiol* **41**, 407-422, https://doi.org/10.1007/s002650050402 (1997).

9 Bell, W. J., Gorton Jr., R. E., Tourtellot, M. K., Breed, M. D. Comparison of male agonistic behavior in five species of cockroaches. *Insect Soc* **26**, 252-263, https://doi.org/10.1007/BF02223804 (1979).

10 Gorton, R. E., Colliander, K. G. & Bell, W. J. Social behavior as a function of context in a cockroach. *Anim Behav* **31**, 152-159, https://doi.org/10.1016/S0003-3472(83)80183-3 (1983).

11 Montagner, H. a. G., G. in *The biology of social insects (M.D. Breed, C.D. Michener and M.E. Evans, Eds.*) 302-306 (Westview Press, 1982).

12 Lenoir, A. An informational analysis of antennal communication during trophallaxis in the ant *Myrmica rubra* L. *Behav Process* **7**, 27-35, https://doi.org/10.1016/0376-6357(82)90050-X (1982).
